# A Pharmacovigilance Analysis of Ocular Adverse Events Associated with GLP-1 Receptor Agonists

**DOI:** 10.3390/jcm15062464

**Published:** 2026-03-23

**Authors:** Abdullah Virk, Karen Allison

**Affiliations:** 1 College of Medicine-Phoenix, University of Arizona, Phoenix, AZ 85004, USA; azvirk@arizona.edu; 2 Flaum Eye Institute, University of Rochester, Rochester, NY 14642, USA

**Keywords:** FAERS, pharmacovigilance, GLP-1 receptor agonists, ocular side effects, diabetic medication ocular toxicity

## Abstract

**Background/Objectives:** Glucagon-like peptide-1 receptor agonists (GLP-1 RAs) are increasingly prescribed for type 2 diabetes in addition to other conditions such as obesity. As their use expands, understanding potential ocular safety signals is important, particularly in populations already at risk for diabetic eye disease. The aim of this study is to identify potential pharmacovigilance safety signals for ocular adverse events (AEs) related to GLP-1 RA medications to better inform future clinical practice. **Methods:** This study utilized the publicly available FDA Adverse Event Reporting System (FAERS) to obtain AE reports related to exenatide, tirzepatide, dulaglutide, liraglutide, and semaglutide from 2005 to 2024. Reports were categorized by demographic and geographic variables. Disproportionality analysis using reporting odds ratios (RORs) was performed to detect potential safety signals. Year-over-year trends in the proportional representation of each drug were also assessed through linear regression and time series plots. **Results:** Ocular AEs represented 3.61% of all GLP-1 RA related reports. Median age was 63 years, and 62.6% of reports involved female patients. Exenatide accounted for 33.61% of ocular AEs but showed a significant annual decline in reporting (–5.15% per year, *p* < 0.001). Semaglutide (31.37%) and tirzepatide (12.19%) demonstrated significant year-over-year increases in proportional reporting (2.23% and 0.79% per year, respectively; both *p* < 0.05), consistent with rapid uptake in clinical practice. Semaglutide demonstrated a modestly elevated ROR (1.46), while tirzepatide showed a low ROR (0.42), though this likely reflects shorter post-marketing exposure rather than lower clinical risk. The most frequently reported events were visual impairment, followed by vision blurred, cataract, and blindness. **Conclusions:** This pharmacovigilance analysis identifies potential ocular AE signals associated with GLP-1 RAs, particularly semaglutide. While semaglutide showed a statistically significant disproportional reporting signal for ocular AEs, the absence of exposure denominators, comparator groups, and the susceptibility of FAERS to reporting bias means these findings are hypothesis-generating rather than causal. Clinicians should remain vigilant and consider eye care referrals when indicated. Further research is needed to validate these associations and clarify underlying mechanisms.

## 1. Introduction

Type 2 diabetes mellitus (T2DM) is a chronic metabolic disorder that is estimated to affect more than 590 million people around the world by 2035, with an estimated global economic burden of more than USD 2.1 trillion by 2030 [1,2]. According to the Centers for Disease Control and Prevention (CDC), more than 38 million Americans have diabetes, with about 90–95% having T2DM. Generally, T2DM is considered to be associated with adults over the age of 45 years, but an increasing number of children are being affected [1,3]. T2DM is characterized by a combination of metabolic abnormalities, including insulin resistance in peripheral tissues, impaired insulin secretion from pancreatic β-cells, dysregulated glucagon secretion from α-cells, and progressive β-cell dysfunction and loss. These disturbances contribute to chronic hyperglycemia and may lead to multisystem complications [1,4]. Risk factors for T2DM include having a family history, being physically inactive, prediabetic, or overweight. Risk of T2DM increases for those over the age of 45 years and for African Americans, Hispanics or Latinos, American Indian, and Alaskan Native populations [3]. T2DM can affect various regions of the body such as the heart, kidney, or eyes. Ocular effects of diabetes include increased risk of diabetic retinopathy, macular edema, diabetic papillopathy, glaucoma, cataracts, various ocular surface diseases, and neovascularization inside the eye that further increases risk of secondary diseases [5]. Oftentimes, these conditions can result in the irreversible loss of vision and subsequent reduction in quality of life of a patient. Hence, it is recommended that patients with diabetes or prediabetes regularly follow up with eye care professionals to prevent disease progression and allow for earlier interventions [6].

There is a plethora of treatment options for diabetes such as lifestyle modifications, bariatric or metabolic surgery, and pharmacologic therapies (Table 1). Medical therapy includes drugs such as Glucagon-like peptide-1 receptor agonists (GLP-1 RAs), sulphonylureas, and biguanides (Metformin) amongst many others [4]. GLP-1 RAs include dulaglutide, liraglutide, semaglutide, exenatide, and tirzepatide (dual GLP-1 and glucose-dependent insulinotropic polypeptide receptor agonist) [7]. GLP-1 is known as an incretin hormone and acts on β and α-cells in the pancreas to increase insulin production while suppressing glucagon secretion [1,4]. β cells are pancreatic cells that produce insulin, a hormone secreted to lower blood sugar levels, while α-cells are pancreatic cells that produce glucagon, a hormone secreted to prevent blood sugar levels from falling too low. Moreover, studies have shown that GLP-1 also inhibits β-cell death and induces β-cell proliferation [7,8,9]. However, since the process of insulin production in T2DM becomes disrupted or absent, GLP-1 RA medications are utilized to treat patients by mimicking the effect of GLP-1.

GLP-1 RAs are most commonly administered subcutaneously but have some oral formulations as well [7]. These medications are also often used to assist with weight management and obesity due to the ability of GLP-1 to suppress appetite by delaying gastric emptying and gut motility [12,13]. Additionally, studies have found cardiovascular risk reduction due to GLP-1 medication use as well [14,15]. However, GLP-1 RAs have various contraindications and an increased risk of gastrointestinal disorders, hypotension, syncope, arthritic disorders, nephrolithiasis, interstitial nephritis, and drug-induced pancreatitis [16,17]. Due to the rising rates of T2DM and obesity in the US, these medications have rapidly grown in usage over the past decade, with monthly rates of increase in usage being 83.9% of Ozempic (semaglutide), 119.2% for Wegovy (semaglutide), 84.8% for Rysbelsus (semaglutide), 53.3% for Saxenda (liraglutide), 78.8% for Trulicity (dulaglutide), and 254.3% for Mounjaro (tirzepatide) during the first 12 months of availability [18]. With the growing usage of GLP-1 RAs worldwide, there is limited published research that analyzes the ocular side-effects of these drugs.

This study utilized the FDA Adverse Event Reporting System (FAERS), which is a publicly available database that contains adverse events (AEs) reports to the U.S. Food and Drug Administration (FDA) from healthcare professionals, consumers, and manufacturers. FAERS was designed to assist the FDA with post-marketing safety surveillance of drugs and therapeutic biological products [19]. Among available medications, glucagon-like peptide-1 receptor agonists have gained prominence due to their glucose-dependent insulinotropic effects, suppression of glucagon secretion, promotion of β-cell survival, and additional cardiovascular and weight management benefits. Despite the widespread usage of GLP-1 RAs, data regarding their ocular side effects are sparse. The primary objective of this study is to identify potential pharmacovigilance safety signals for ocular adverse events related to GLP-1 RAs to assist clinicians in their decision making and risk assessment regarding these medications. Given the rapidly growing global population of patients with diabetes, along with the increasing use of GLP-1 receptor agonists, even uncommon medication related side effects may affect a substantial number of individuals and have a meaningful impact on quality of life. Patients with diabetes are already at risk for ocular disease and are often prescribed these medications on a long-term regime. Hence, it is critical that clinicians learn to identify and characterize any ocular effects associated with GLP-1 RAs in order to prevent undesired visual symptoms. Additional awareness of medications that have an increased likelihood of ocular AEs can also help educate patients and guide future research along with clinical practice.

## 2. Methods

This study utilized the publicly available FDA Adverse Event Reporting System (FAERS) database with the search terms exenatide, tirzepatide, dulaglutide, liraglutide, and semaglutide. Using R statistical analysis software (version 4.3.2) and Microsoft Excel, cases within our study period of 2005 through 2024 were isolated and sorted by medication or adverse event. AE reports were categorized by variables such as country of origin and gender. Deduplication by case ID was applied to summary level data. In contrast, reports related to individual GLP-1 medications that had multiple ocular AEs were treated as separate cases to ensure all events were included. Disproportionality in reporting was evaluated using reported odds ratios (RORs) with 95% confidence intervals. The ROR analysis was based on a 2 × 2 contingency table, which is shown in Table 2. The ROR was calculated using the formula (a/b)/(c/d), which is equivalent to (a × d)/(b × c). A positive signal was defined as the lower limit of 95% CI being >1 and *N* > 3, indicating a drug could have an association with an adverse event. Yearly trends in the proportional representation of each drug were also assessed through linear regression and time series plots.

## 3. Results

In the study period from 2005 to 2024, there were a total of 283,552 adverse events from GLP-1 RAs (exenatide, dulaglutide, tirzepatide, semaglutide, and liraglutide) in FAERS. Out of the total AE reports for these medications, 3.61% (10,237 cases reported) were ocular AEs. The median age for patients in reports for GLP-1 ocular AEs was 63 while 62.6% of reported cases were females (Table 3).

Exenatide comprised 33.61% of total GLP-1 ocular AE reports in the study period, followed by dulaglutide (33.14%), semaglutide (31.37%), tirzepatide (12.19%), and liraglutide (12.03%). Although exenatide was the GLP-1 medication with most AE reports, the median year of those reports was 2013, with 2006 being the year with the maximum number of reports. Notably, tirzepatide and semaglutide have the most recent surge in reports, with 2024 being the year with maximum ocular AE reports and the median year of total ocular AE reports being 2024 and 2023, respectively. Overall, semaglutide showed a higher reporting frequency of ocular AEs compared to other drugs in the FAERS database (ROR = 1.46, 95% CI: 1.40–1.52). All other medications had ROR values below 1, indicating no disproportional reporting of ocular AEs. The US was the primary source of all ocular-related AE reports for GLP-1 RAs, with dulaglutide (94.11%) and tirzepatide (90.87%) having the highest percentages of ocular AE reports and exenatide having the lowest percentage from the United States (51.53%). These results represent reporting signals rather than any estimates of causation, particularly because FAERS does not provide data on the total number of patients exposed to a drug.

**Figure 1:**
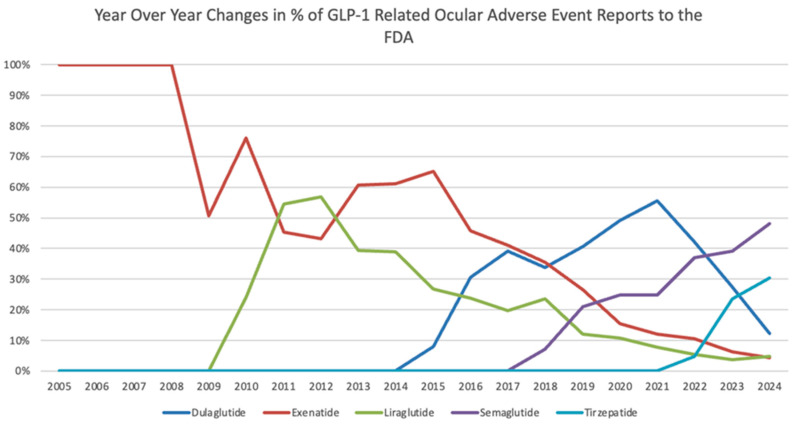
Year-over-year changes in percent of ocular adverse event reports to the FDA related to GLP-1 receptor agonists.

**Figure 2:**
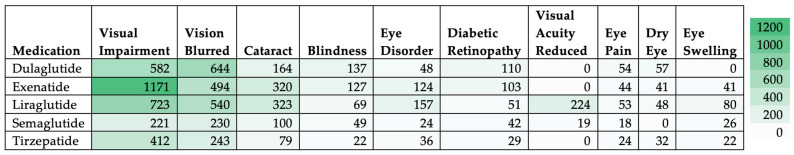
Heatmap showing the top 10 ocular adverse events reported for GLP-1 receptor agonists in FAERS.

Year-over-year changes in the proportional representation of each medication regarding total ocular AE reports within the study period can be found in Figure 1. Linear regression was performed to determine statistical significance of change in the proportional representation of GLP-1 RAs among related ocular AEs from 2005 to 2024. Exenatide showed a steady decline in ocular AE reports over the study period with a statistically significant (*p* < 0.001) decrease in proportional reporting with an average annual decrease of 5.15% from 2005 to 2024 (95% CI: −6.14, −4.17). Dulaglutide and semaglutide showed a statistically significant (*p* < 0.001) annual increase during the study period of 2.62% and 2.23%, respectively. Tirzepatide also exhibited a statistically significant (*p* = 0.01) increase of 0.79% annually. However, liraglutide did not display a statistically significant average percent change per year (Table 3). The types of ocular AE reports are shown in Figure 2. Overall, “visual impairment” and “vision blurred” were the top 2 ocular AE report types for all medications, followed by cataract and blindness (Figure 2).

The majority of ocular AE reports due to GLP-1 RAs originated from the United States (US) by a large margin, followed by the United Kingdom (UK) and Canada (Table 4). Semaglutide was present in the top two GLP-1 RAs for the US, UK, and Canada. Interestingly, although dulaglutide was the most reported GLP-1 RA in the United States, it was the fifth most reported GLP-1 RA for the UK and the third for Canada.

## 4. Discussion

The results offer an insight into GLP-1 receptor agonists and related ocular adverse events. Although exenatide comprised the highest percentage of total ocular AE reports over the study period (33.14%), newer and increasingly used medications such as semaglutide and tirzepatide exhibited a growth in proportional reports (31.37% and 12.19%, respectively). It is important to note that exenatide had the longest market exposure with FDA approval in April 2005, which might account for the larger proportion of reports [20]. Semaglutide and tirzepatide have grown in popularity recently due to their efficacy in weight loss along with having mild-to-moderate-severity adverse effects [21]. This change in proportional usage and AE reporting of the GPL-1 RAs can be seen in Table 3 and Figure 1, with exenatide having a statistically significant year-over-year decrease in proportional reporting (−5.15%, 95% CI: −6.14, −4.17) over the study period while semaglutide and tirzepatide have a statistically significant increase in ocular AE reports in a recent surge since 2018 and 2022, respectively. This increase in reporting started when the drugs were first approved by the FDA, with semaglutide (Ozempic) being first approved in December 2017 and tirzepatide (Mounjaro) being approved in May 2022 [22,23]. The increased market availability and prescription volume could have influenced the noticeable rise in the ocular AE reports of these drugs. Semaglutide was found to have a statistically significant disproportional reporting signal for ocular AEs (ROR = 1.46, 95% CI: 1.40–1.52). This indicates that semaglutide has a higher association with ocular AE reports compared to all other drugs in FAERS. However, all other GLP-1 medications had ROR values less than 1, indicating no disproportional reporting of ocular AEs compared to other drugs in the FAERS database. This lack of disproportional reporting should not be interpreted as evidence of lower risk, just lack of association. With certain newer medications, such as tirzepatide (ROR = 0.42, 95% CI: 0.39–0.45), there are shorter post-marketing windows and market exposure causing increased susceptibility to reporting patterns and biases. Thus, tirzepatide’s lower ROR value should not be interpreted as lower risk due to its shorter post-marketing period. Future studies should examine longer post-marketing periods to better assess potential associations with ocular adverse effects. Due to most patients having multiple coexisting side effects, with each ocular AE tallied for specific medications, the sum of results for the individual medications is greater than the total results for GLP-1 RAs, which were based on unique report case IDs.

Overall, a substantial number of ocular AEs reported were visual impairment, vision blurred, cataract, blindness, eye disorder, and diabetic retinopathy (Figure 2). Ocular event reports such as cataract, diabetic retinopathy, or visual problems might have just been pre-existing conditions, which were included in the overall list of symptoms experienced by the patient. This is supported by the existing literature, where diabetes patients or patients with existing comorbidities already have an increased risk for diabetic retinopathy, cataract development, and other conditions, such as glaucoma, due to a variety of mechanisms [24]. The AEs identified in this study could be secondary to the underlying disease rather than the medication, which is a critical limitation due to the inability to distinguish the drug-induced event from the natural progression of the diabetic eye disease. However, previous literature has shown that many GLP-1 RAs had strong associations with diabetic retinopathy, with semaglutide having significantly higher odds of ischemic optic neuropathy, diabetic retinopathy, and retina/vitreous damage [25,26]. Moreover, patients with T2DM prescribed GLP-1 RAs are more likely to be seen by eye care professionals compared to other patients such as those prescribed GLP-1 RAs for obesity, resulting in possible differences in reporting patterns based on the patient population [27].

Most ocular AE reports (62.6%) originated from female patients. Responses to GLP-1 receptor agonists can vary depending on the gender, most likely due to the specific hormonal variations [28]. In fact, prior research has found that weight loss is more prominent in females taking GLP-1 RAs, but women are more likely to have medication related adverse events in the gastrointestinal system [28,29]. Still, current data do not entirely explain the mechanisms for the demographic differences, and further studies need to expand on these findings. Although studies on the exact mechanisms of action linking these drugs to ocular side effects are limited, possible mechanisms include decreases in blood sugar levels causing an influx of water from the aqueous humor into the lens as a result of differences in osmotic pressure. This influx in water causes swelling of the lens, resulting in temporary blurred vision until blood sugar is stabilized [30,31]. Symptoms may persist until medication excretion, which is conducted through renal elimination by the kidneys [7]. The time each medication spends in the body can vary with many formulations having different half-lives, which can be seen with drugs such as semaglutide being widely utilized due to its extended half-life [7,32]. Prior research has also highlighted the rare but possible likelihood of systemic hypersensitivity due to GLP-1 medications, which could result in potential secondary conjunctivitis [33,34,35,36]. Changes in vision could also occur due to the rapid initiation of glycemic control, which can precipitate early worsening of diabetic retinopathy. This is another, more indirect, method in which GLP-1 medications, amongst other diabetic treatments, can influence visual acuity and eye health [37]. Nevertheless, additional research is warranted in order to understand the mechanisms underlying various ocular side effects secondary to GLP-1 RA use and the respective demographic differences that are associated.

The vast majority of reports originated from the US, with first-world countries such as the United Kingdom and Canada following. Still, much of the AE data reported to FAERS are from the US rather than foreign countries, especially those in Africa, Asia, and South America [38]. A possible explanation is that the US and other first-world countries have more access to drugs such as GLP-1 RAs and have existing pharmacovigilance systems in place, which could result in a higher proportion of existing AE data originating from those populations. As a result, current data may lack diversity and not apply to populations in different environments and with varying genetic backgrounds [39]. Lastly, the FAERS database is operated by the FDA, which is a United States based agency in the Department of Health and Human Services, resulting in the majority of the dataset containing reports originating from American patients, clinicians, and producers, accounting for the large discrepancy in the geographic distribution of reports [19,40]. As a result, these findings are not globally generalizable and further support development of pharmacovigilance systems in other regions.

### Limitations

There are some limitations to this study, particularly related to the FAERS database. Due to the spontaneous nature of reporting in FAERS, there can be potential for reporting bias, notoriety bias, and Weber effect, where drug AE reporting peaks in the second year after approval and subsequently declines [41]. Additional temporal bias may also be present due to the variability of market exposure of different GLP-1 medications, with exenatide having nearly two decades of reporting and tirzepatide having only a few years. Another limitation is that there is no causal relationship between a medication and a reported AE, especially since patients can have multiple comorbidities and treatment regimens. There is a lack of relevant background information in the reporting to establish any causal relationships [19,42]. Because of this, many of the reported ocular AEs identified in this study may be attributed to the natural progression of diabetic eye disease or preexisting conditions rather than secondary to GLP-1 RAs. The presence of preexisting ocular diseases is an important confounding factor that further limits any causal interpretation of these relationships. Furthermore, the lack of an external comparator group (e.g., SGLT2 inhibitors) further limits this study’s specificity and ability to provide confirmatory evidence of causal relationships. The FAERS database does not provide the total number of patients exposed to medications, preventing any viable calculations on incidence rates. Moreover, the database does not report race or ethnicity data, and certain reports also have missing data regarding country of origin, date of report, gender, or age [19]. As a result of these limitations, all findings should be interpreted as hypothesis-generating rather than causal. Additional research is necessary to establish any causal relationships related to GLP-1 RAs and specific ocular adverse events.

## 5. Conclusions

Type 2 diabetes mellitus (T2DM) remains a significant global health concern, with increasing prevalence across all age groups and substantial systemic and ocular health impacts. Diabetic eye disease continues to be one of the leading causes of preventable blindness worldwide, highlighting the importance of regular ophthalmic screening and early intervention. GLP-1 RAs represent a notable advancement in T2DM management. Their capacity to enhance insulin secretion, lower glucagon levels, and support β-cell preservation directly targets key aspects of T2DM pathophysiology. Additionally, their benefits, including weight loss and cardiovascular protection, have broadened their clinical applications. The results highlight pharmacovigilance signals for ocular adverse events associated with GLP-1 RAs. Semaglutide was the only medication with a statistically significant disproportional reporting signal for ocular adverse events, indicating a higher association with ocular adverse event reports compared to all other drugs in FAERS. These findings suggest that there may be potential ocular side effects of GLP-1 RAs, which include visual impairment or blurry vision amongst others. Clinicians should stay vigilant regarding patients with GLP-1 RA usage and refer to appropriate eye care providers if needed. Through improved understanding of adverse events due to these increasingly used GLP-1 RAs, healthcare providers can ensure the best outcomes for their patients. Future research should focus on more diverse and granular databases and clinical trials to build upon and validate these findings in addition to elucidating the exact mechanism of action for the ocular side effects related to GLP-1 receptor agonists.

## Figures and Tables

**Table 1:** Current treatments for type 2 diabetes [10,11].

**Oral Medications**	**Injectable Medications**	**Insulin**	**Lifestyle Modifications**
Biguanides (e.g., Metformin)	GLP-1 Agonists	Basal insulin	Consuming healthy meals and beverages
Sulfonylureas (e.g., glyburide)	Amylin Analog (e.g., pramlintide acetate)	Combination basal insulin/GLP-1 agonist	Reduced sedentary lifestyle
Meglitinides (e.g., repaglinide)	Insulin	Bolus insulin	Increased physical activities
Thiazolidinediones (e.g., pioglitazone)		Rapid-acting insulin	Stopping smoking
Sodium-glucose cotransporter 2 (SGLT-2) inhibitors (e.g., canagliflozin)		Short-acting insulin	Reducing alcohol intake
Dipeptidyl peptidase-4 inhibitors (e.g., sitagliptin)		Inhaled insulin	
α-Glucosidase inhibitors (e.g., acarbose)		Premixed insulin	
Bile acid sequestrant (e.g., colesevelam)			
Dopamine receptor agonist (e.g., bromocriptine)			

**Table 2:** Two-by-two contingency table for disproportionality analysis.

	Ocular Adverse Events	Non-Ocular Adverse Events
Drug of Interest	a	b
All FAERS Medications	c	d

**Table 3:** GLP-1 medication ocular adverse event report characteristics.

Drug Name	Total Adverse Events	Ocular Adverse Events	% of Total Adverse Events	US Adverse Ocular Events	% Total Ocular Adverse Events	ROR (95% CI)	Maximum Ocular Reports in a Year	Year with Maximum Ocular Reports	Median Year (Q1–Q3)	Reported Female	% Female Drug Specific	Age Median (Q1–Q3)	Average % Change per Year (95% CI), *p* Value
Exenatide	78,994	3441	4.36%	1773	51.53%	0.75 (0.72–0.78)	446	2006	2013 (2007–2019)	2222	64.57%	63 (56–69.5)	−5.15 (−6.14, −4.17), *p* < 0.001
Dulaglutide	69,987	3393	4.85%	3193	94.11%	0.89 (0.86–0.92)	781	2021	2021 (2020–2022)	2079	61.27%	63.5 (57–72)	2.62 (1.54, 3.71), *p* < 0.001
Tirzepatide	56,943	1248	2.19%	1134	90.87%	0.42 (0.39–0.45)	847	2024	2024 (2023–2024)	820	65.71%	58 (49–68)	0.79 (0.21, 1.37), *p* = 0.01
Semaglutide	40,087	3212	8.01%	2360	73.47%	1.46 (1.40–1.52)	1341	2024	2023 (2021–2024)	1963	61.11%	63 (55–71)	2.23 (1.47, 3.00), *p* < 0.001
Liraglutide	37,541	1232	3.28%	771	62.58%	0.62 (0.58–0.66)	150	2018	2018 (2015–2021)	752	61.04%	62 (53–68)	−0.09 (−1.61, 1.43), *p* = 0.91
GLP-1	283,552	10,237	3.61%	7630	74.53%	0.8 (0.78–0.82)	2339	2024	2021 (2018–2023)	6408	62.60%	63 (55–70)	

**Table 4:** Top 5 drugs for each of the top 3 countries associated with adverse ocular reports related to GLP-1 medications from 2005 to 2024.

1. United States			2. United Kingdom			3. Canada		
Drug Name	Ocular Adverse Events Reports	% of Total Country Ocular Adverse Events Reports	Drug Name	Ocular Adverse Events Reports	% of Total Country Ocular Adverse Events Reports	Drug Name	Ocular Adverse Events Reports	% of Total Country Ocular Adverse Events Reports
Dulaglutide	3193	34.59%	Semaglutide	91	40.27%	Semaglutide	129	60.28%
Semaglutide	2360	25.57%	Tirzepatide	57	25.22%	Liraglutide	78	36.45%
Exenatide	1773	19.21%	Exenatide	45	19.91%	Dulaglutide	5	2.34%
Tirzepatide	1134	12.28%	Liraglutide	19	8.41%	Exenatide	2	0.93%
Liraglutide	771	8.35%	Dulaglutide	14	6.19%	Tirzepatide	0	0.00%

## Data Availability

The data presented in this study are openly available from the FDA Adverse Event Reporting System (FAERS) at https://www.fda.gov/drugs/fdas-adverse-event-reporting-system-faers/fda-adverse-event-reporting-system-faers-public-dashboard (accessed on 31 January 2025).
